# Osteogenic Potential of a Three‐Phase Strontium‐ and Silicon‐Doped Tricalcium Silicate Cement on Dental Pulp Stem Cells: An In Vitro Study

**DOI:** 10.1002/cre2.70362

**Published:** 2026-05-03

**Authors:** Nahid Nasrabadi, Ehsan Saburi, Hossein Bagheri, Atekeh Hosseinpour, Mahsa Ghorbani, Pooya Saeedi

**Affiliations:** ^1^ Private Practice Mashhad Iran; ^2^ Department of Medical Genetics and Molecular Medicine, Faculty of Medicine Mashhad University of Medical Sciences Mashhad Iran; ^3^ Dental Materials Research Center, School of Dentistry Mashhad University of Medical Sciences Mashhad Iran; ^4^ Dental Research Center Mashhad University of Medical Sciences Mashhad Iran

**Keywords:** biocompatible materials, bone substitutes, calcium silicates, dental pulp stem cells, hydroxyapatites, osteogenesis

## Abstract

**Objective:**

This study aimed to evaluate the osteogenic potential of a novel three‐phase cement composed of strontium‐doped hydroxyapatite (Sr‐HA), silicon‐doped hydroxyapatite (Si‐HA), and tricalcium silicate (C_3_S) on dental pulp stem cells (DPSCs) in vitro.

**Methods:**

Sr‐HA, Si‐HA, and C_3_S were synthesized via sol–gel methods, combined with anhydrous calcium sulfate to produce a three‐phase cement (3P cement), and characterized using scanning electron microscopy and X‐ray diffraction. DPSCs were cultured on three dilutions of 3P cement and compared with mineral trioxide aggregate (MTA), Biodentine (BDNT) cement, and a control medium. Cell viability and proliferation were assessed using the MTT assay, apoptosis was evaluated by Annexin V/PI staining, and osteogenic differentiation was analyzed through alkaline phosphatase (ALP) activity, calcium deposition, and Alizarin Red staining on Days 7 and 14.

**Results:**

The 3P cement exhibited excellent biocompatibility, with the highest DPSC proliferation observed at the 1/5 dilution. Apoptosis rates were minimal and comparable to controls. DPSCs cultured on 3P cement demonstrated significant increases in ALP activity, calcium deposition, and mineralized nodule formation compared to the control (*p* < 0.05). The osteogenic response was comparable to, or in some measures superior to, MTA and BDNT cement.

**Conclusions:**

The three‐phase Sr‐ and Si‐doped tricalcium silicate cement supports DPSC viability and promotes osteogenic differentiation, indicating its potential as a bioactive bone substitute. Further preclinical and in vivo studies are warranted to validate its regenerative efficacy in clinical applications.

## 1 Introduction

Bone defects caused by conditions such as advanced periodontitis, trauma, or congenital anomalies present significant clinical challenges, often leading to functional and aesthetic problems as well as high treatment costs. The primary goal in managing these defects is to promote bone regeneration and restore both structure and function (Villar and Cochran 2010). Bone grafting remains one of the most widely used approaches to achieve this goal and can be performed using natural scaffold materials, including autografts, allografts, and xenografts, or synthetic materials known as alloplastic grafts (Reynolds et al. 2004).

Autologous bone grafts are considered the gold standard due to their excellent biocompatibility and strong regenerative potential. However, they are limited by drawbacks such as donor site morbidity, postoperative discomfort, the need for a secondary surgical procedure, and the restricted amount of graft material that can be harvested (AlGhamdi et al. 2010; Sheikh et al. 2017). Allografts and xenografts overcome some of these issues but have potential disadvantages, including variable dimensional stability over time, the risk of tissue contamination, and the possibility of immunological reactions or cross‐infection (Turco et al. 2018; Q. Zhang et al. 2019).

Alloplastic materials have been developed to address these challenges and are widely used due to their high biocompatibility, unlimited availability, and consistent quality. They are produced in various forms, such as blocks, granules, powders, and injectable cements. Hydroxyapatite (HA), tricalcium phosphate (TCP), and bioactive glasses are among the most commonly used synthetic materials in periodontal bone regeneration (AlGhamdi et al. 2010). While these materials are biocompatible, their limited biodegradability and osteoinductive capacity restrict their ability to achieve complete bone regeneration (Sheikh et al. 2017; Q. Zhang et al. 2019)

Recent research has focused on silicate‐based bioceramics, which are bioactive, resorbable, and capable of stimulating robust bone repair. Incorporating silicon (Si) ions into the HA structure creates a modified apatite that closely resembles the inorganic component of natural bone. Silicon has been shown to stimulate osteogenic differentiation of mesenchymal stem cells (MSCs) by enhancing alkaline phosphatase (ALP) activity and promoting the expression of osteogenesis‐related genes and proteins (Ana et al. 2018; Shi et al. 2015; Wu and Chang 2013). Similarly, strontium (Sr) ions can substitute for calcium in the HA lattice, stimulating bone formation while simultaneously inhibiting bone resorption. Sr promotes osteoblastic differentiation, increases cell proliferation, and reduces apoptosis, thereby contributing to improved bone remodeling (Jiménez et al. 2019; Pors Nielsen 2004).

Calcium silicate (CS)‐based biomaterials have also gained attention due to their favorable mechanical properties, controlled biodegradability, and inherent osteogenic potential (Cao et al. 2018; Huang et al. 2019; L. Zhang et al. 2016). Tricalcium silicate (C_3_S), in particular, is widely used in orthopedic and dental applications because of its injectability, self‐setting capability, and ability to adapt to irregularly shaped defects (Liu et al. 2019). These materials release bioactive silicon ions that play a key role in bone tissue regeneration (Cao et al. 2018; Carlisle 1981).

Dental pulp stem cells (DPSCs), a type of MSCs, have demonstrated strong potential for bone tissue engineering due to their ability to differentiate into osteoblasts under appropriate stimuli (Davies et al. 2015; Monterubbianesi et al. 2019; Tóth et al. 2020). Several studies have explored the osteogenic potential of bioactive cements on DPSCs (Bhandi et al. 2021; Huo et al. 2021). Combining different bioactive phases may enhance the regenerative capacity of these materials.

Considering the individual benefits of Si‐HA, Sr‐HA, and C_3_S, this study aimed to evaluate the osteogenic induction potential of a novel three‐phase cement composed of these components using DPSCs. The findings may contribute to the development of more effective synthetic graft materials for periodontal and craniofacial bone regeneration.

## 2 Methods and Materials

### 2.1 Samples Preparation

Sr‐substituted hydroxyapatite (Sr‐HA) was synthesized using a sol–gel method in a water‐alcohol solution, with Sr^2+^ ions replacing Ca^2+^ in the HA structure. Briefly, a mixture of SrCl_2_ (0.05 mol) and CaCl_2_ (0.45 mol) was dissolved in 200 mL of deionized distilled water, while NaH_2_PO_4_ (0.3 mol) was dissolved separately in another 200 mL of deionized water. A 1 N NaOH solution was added to maintain the pH at 10. The SrCl_2_/CaCl_2_ solution was slowly added to the phosphate solution over 1 h, followed by further adjustment of pH to 10 using 1 N NaOH. The mixture was stirred continuously for 72 h at 25°C. The resulting suspension was centrifuged at 2504 g to separate the liquid phase, and the precipitate was dried at room temperature.

To complete crystallization and eliminate residual nitrates, the dried powder was sintered at 800°C for 2 h and rapidly cooled to room temperature at a rate of 10°C/min. The sintered material was mechanically ground and sieved through a 45‐µm mesh.

Si‐substituted hydroxyapatite (Si‐HA) was prepared using a similar sol–gel process, where SiO_3_^2−^ replaced PO_4_^3^^−^ in the HA structure. Tetraethyl orthosilicate (TEOS, 0.02 mol) was dissolved in 100 mL of an ethanol–water solution, and NaH_2_PO_4_ (0.28 mol) was dissolved in 100 mL of deionized water. The two solutions were combined, and the pH was adjusted to 10 using 1 N NaOH. Separately, CaCl_2_ (0.5 mol) was dissolved in 200 mL of deionized water and gradually added to the Si‐ and phosphate‐containing solution over 1 h. The mixture underwent the same centrifugation, drying, sintering, grinding, and sieving steps as described for Sr‐HA.

Tricalcium silicate (C_3_S) was synthesized using a sol–gel method. TEOS (0.5 mol) was dissolved in 200 mL of a nitric acid–water solution (HNO_3_ as the catalyst) to initiate hydrolysis. Ca(NO_3_)_2_ (1.5 mol) was then added, and the mixture was stirred at 80°C until a gel was formed. The gel was oven‐dried at 120°C and subsequently sintered at 1200°C for 10 h. The final material was pulverized and sieved to a particle size of 45 µm.

The three synthesized powders were combined at a ratio of 25 wt.% Sr‐HA, 25 wt.% Si‐HA, and 50 wt.% C_3_S. This mixture was blended with anhydrous CaSO_4_ in a 1:1 weight ratio to generate porosity, resulting in a final composition of 12.5 wt.% Sr‐HA, 12.5 wt.% Si‐HA, 25 wt.% C_3_S, and 50 wt.% CaSO_4_.

For comparison, mineral trioxide aggregate (MTA; Angelus, Brazil) and Biodentine (BDNT; Septodont, the United States) were used as commercial reference materials.

### 2.2 Morphological Characterization

The morphology and crystalline structure of the synthesized three‐phase cement have been previously characterized using scanning electron microscopy (SEM) and X‐ray diffraction (XRD), as reported by Parisay et al. (2022). The present study focused exclusively on evaluating the biological performance of the previously characterized material.

### 2.3 Cell Culture

The DPSC line was obtained from the Royan Institute (IBRC C10266; Tehran, Iran). Cells were cultured in Dulbecco's Modified Eagle Medium (DMEM; Sigma, Germany) supplemented with 10% fetal bovine serum (FBS) and 1% penicillin–streptomycin (both from Merck, Germany). The medium pH was adjusted to 7.4 using HCl or NaOH (Merck, Germany). Cultures were maintained in a humidified incubator (Memmert, Germany) at 37°C and 5% CO_2_.

The culture medium was replenished every 2 days or upon color change. Once the cells reached approximately 80% confluence, they were detached using trypsin (Gibco, the United States) and incubated for 5 min at 37°C. Trypsin activity was neutralized by adding 10% FBS, and the cell suspension was processed using an automated cell counter (Bio‐Rad, the United States).

For cryopreservation, a solution containing 40% DMEM, 50% FBS, and 10% dimethyl sulfoxide (DMSO; Merck, Germany) was used. Cells were initially frozen at −80°C for 24 h before long‐term storage in liquid nitrogen. Thawing was performed under sterile conditions using a laminar flow hood (Jal Tajhiz, Iran). After gradual thawing and gentle pipetting, DMEM containing 20% FBS was added, and the cells were transferred to flasks for further culture.

### 2.4 Assay Setup and Exposure Conditions

DPSCs were used between Passages 3 and 6 for all experiments. Cells were seeded at densities optimized in preliminary experiments to achieve approximately 70%–80% confluence at the time of treatment. For MTT assay, cells were seeded in 96‐well plates at approximately 5 × 10^3^ to 1 × 10^4^ cells per well and allowed to attach for 24 h prior to exposure to material extracts. Cell viability was assessed after 24, 48, and 72 h of treatment. For apoptosis analysis (Annexin V‐FITC/PI), cells were seeded in 6‐well plates at approximately 1–2 × 10^5^ cells per well and exposed to material extracts for 24 h prior to flow cytometric evaluation. For the ALP activity assay, cells were seeded in 24‐well plates at approximately 2–4 × 10^4^ cells per well. Following osteogenic induction and extract treatment, ALP activity was measured at Days 7 and 14. And for calcium quantification and Alizarin Red staining, cells were seeded in 24‐well plates at approximately 2–4 × 10^4^ cells per well. Mineralization assays were performed at Days 7 and 14 following osteogenic induction and extract exposure. All experiments were performed in triplicate and repeated in three independent experimental runs.

### 2.5 Cement Preparation for Cell Culture

For experimental testing, the synthesized cement, MTA, and BDNT were prepared immediately before use. The three‐phase cement powder was mixed with distilled water at a 1:1 weight ratio. MTA and BDNT were prepared according to the manufacturer's instructions. The freshly mixed cements were incubated in complete culture medium at 37°C for 24 h to obtain the conditioned extracts. The extracts were filtered (0.22 µm) to remove particulate matter and then applied to DPSC cultures at the designated concentrations. Experimental groups included: (1) negative control (complete culture medium), (2) reference material groups (MTA and BDNT extracts), and (3) experimental group (three‐phase cement extracts).

### 2.6 MTT and Apoptosis Assay

Cell viability and metabolic activity were evaluated using the MTT assay. DPSCs were seeded in 96‐well plates and exposed to the designated material extracts. At the specified time points (Days 1, 3, 5, and 7), MTT solution (final concentration: 5 mg/mL; Sigma‐Aldrich, the United States) was added to each well and incubated for 4 h at 37°C in a humidified atmosphere containing 5% CO_2_. The formed formazan crystals were dissolved in dimethyl sulfoxide (DMSO; Sigma‐Aldrich, the United States), and optical density (OD) was measured at 570 nm using a microplate reader (BioTek Instruments, the United States). Cell viability was expressed as a percentage relative to the negative control at Day 1 (set as 100%).

Apoptosis was assessed using an Annexin V‐FITC Apoptosis Detection Kit (Sigma‐Aldrich, St. Louis, Missouri, the United States; Cat. No. APOAF) according to the manufacturer's instructions. DPSCs were prepared as a suspension and incubated with 1 mL of the respective material extract for 24 h. For the negative control, cells were incubated with culture medium alone, and for the positive control, cells were treated with mitomycin C for 24 h. Following incubation, cells were washed twice with cold phosphate‐buffered saline (PBS) and resuspended in Annexin V binding buffer. A total of 100 µL of the cell suspension was transferred into a 5‐mL flow cytometry tube, followed by the addition of 5 µL Annexin V‐FITC and 10 µL propidium iodide (PI). Samples were gently vortexed and incubated for 15 min at room temperature in the dark. Subsequently, 400 µL of binding buffer was added, and samples were analyzed within 1 h using a flow cytometer. Apoptotic populations were quantified using FlowJo software (version 7.6), and total apoptosis (%) was calculated as the sum of early and late apoptotic cells. Apoptosis measurements were performed at Days 1, 3, and 7 as independent 24‐h exposure experiments. Each condition was tested in triplicate.

### 2.7 Osteogenic Differentiation Evaluation

#### 2.7.1 Alkaline Phosphates Activity

ALP activity was quantified using a commercial ALP assay kit (PAAD Co., Tehran, Iran). Total protein was extracted by adding RIPA lysis buffer and sonicating the samples for 40 s on ice. After centrifugation at 4000 g for 15 min at 4°C, the supernatant was collected. ALP activity was determined by reading absorbance at 410 nm using an ELISA plate reader (BioTek Instruments, the United States).

#### 2.7.2 Calcium Content Assay

Calcium deposition was evaluated on Days 7 and 14 using a calcium content assay kit (PAAD Co., Tehran, Iran). Samples were treated with 0.6 N HCl (Merck, Germany) and centrifuged at 352 g for 15 min at 4°C, and the OD was measured at 570 nm. Calcium concentration was calculated using a standard curve generated from known calcium dilutions.

#### 2.7.3 Alizarin Red S Staining

Mineralized nodules were visualized on Days 7 and 14. Cells were rinsed with PBS, fixed with 10% formaldehyde for 15 min, and stained with 40 mmol/L Alizarin Red S (Cyto Matin Gene, Iran; pH 4.1) for 20 min. Excess dye was removed by washing with distilled water, and the stained cultures were imaged using an inverted microscope.

### 2.8 Statistical Analysis

All experiments were performed in triplicate, and data were reported as mean ± standard deviation (SD). Statistical comparisons were conducted using two‐way analysis of variance (ANOVA) followed by Tukey's post hoc test. A *p* value of < 0.05 was considered statistically significant. Analyses were performed using GraphPad Prism 6 (Dotmatics, the United States).

## 3 Results

### 3.1 Biocompatibility Characterization

#### 3.1.1 MTT Assay

Cell viability and proliferation of dental pulp stem cells (DPSCs) cultured on three dilutions of the experimental cement (1, 1/2, and 1/5; referred to as Test_1_, Test_1/2_, and Test_1/5_, respectively) were compared with MTA, a negative control, and a positive control over 7 days.

Two‐way ANOVA revealed a time‐dependent increase in DPSC viability in all groups except the positive control. On Day 3, the viability of Test_1/5_ was significantly higher than Test_1_ (*p* < 0.05), and this difference was even greater by Day 7 (*p* < 0.001).

**Figure 1:**
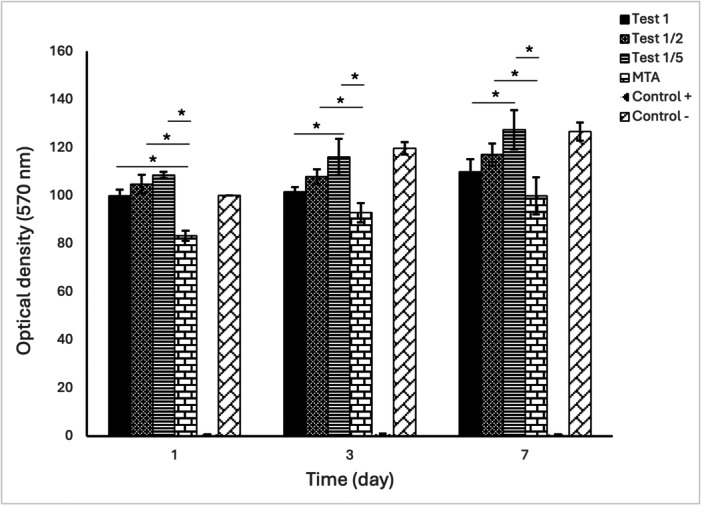
MTT assay showing viability and proliferation of DPSCs cultured on three dilutions of 3P cement, MTA, negative control, and positive control over 7 days.

On Day 1, the Test_1_ group demonstrated significantly higher viability than MTA (*p* < 0.05). Across all time points, both Test_1/2_ and Test_1/5_ exhibited significantly greater viability than MTA. By Day 7, the highest proliferation was observed in Test_1/5_, followed by Test_1/2_ and Test_1_ (Figure 1 and Supplementary Table S1).

#### 3.1.2 Apoptosis

Apoptosis was evaluated to assess potential cytotoxicity in DPSCs exposed to the different material extracts. Two‐way ANOVA revealed a significant interaction between treatment group and time (*p* < 0.05). On Days 1 and 3, the undiluted 3P‐cement extract (Test_1_) demonstrated significantly higher apoptosis compared with all other groups (*p* < 0.05). The 1/2 dilution (Test_1/2_) also showed significantly higher apoptosis than the negative control at these early time points (*p* < 0.05).

**Figure 2:**
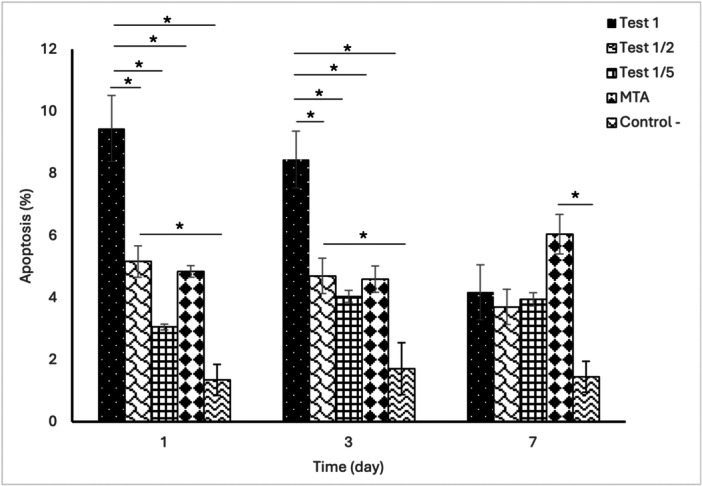
Apoptosis assay illustrating mean apoptosis rates of DPSCs cultured on three dilutions of 3P cement, MTA, and a negative control over 7 days.

By Day 7, apoptosis levels in the 3P‐cement groups decreased and were comparable to the control group. In contrast, MTA exhibited significantly higher apoptosis compared with the negative control at Day 7 and remained elevated across all evaluated time points (Figure 2 and Supplementary Table S2).

### 3.2 Osteogenic Differentiation

The osteogenic potential of the cements was assessed through ALP activity, calcium deposition, and Alizarin Red staining. Four groups were analyzed: 3P cement, MTA, BDNT, and control, at Days 7 and 14 after induction of osteogenic differentiation. Shapiro–Wilk testing confirmed normal data distribution.

#### 3.2.1 ALP Activity and Calcium Content

Two‐way ANOVA indicated a significant effect of both time and cement type on ALP activity and calcium deposition (*p* < 0.001). Independent *t*‐tests showed that, for all groups, both parameters were significantly higher on Day 14 compared to those on Day 7.

On Day 7, the control group had significantly lower ALP activity and calcium content than all other groups (*p* < 0.05). BDNT exhibited significantly higher ALP activity than all other groups (*p* < 0.05), while there was no difference between MTA and 3P Cement. Calcium content did not significantly differ among the experimental cements.

**Figure 3:**
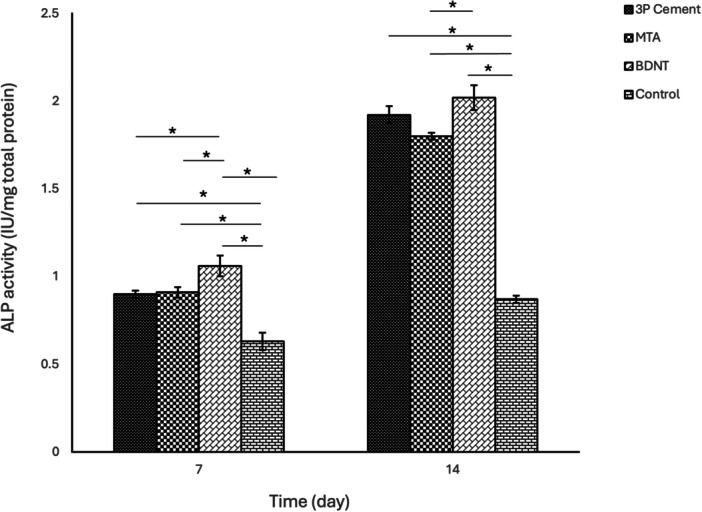
Mean ALP activity with standard deviations and 95% confidence intervals for study groups on Days 7 and 14.

**Figure 4:**
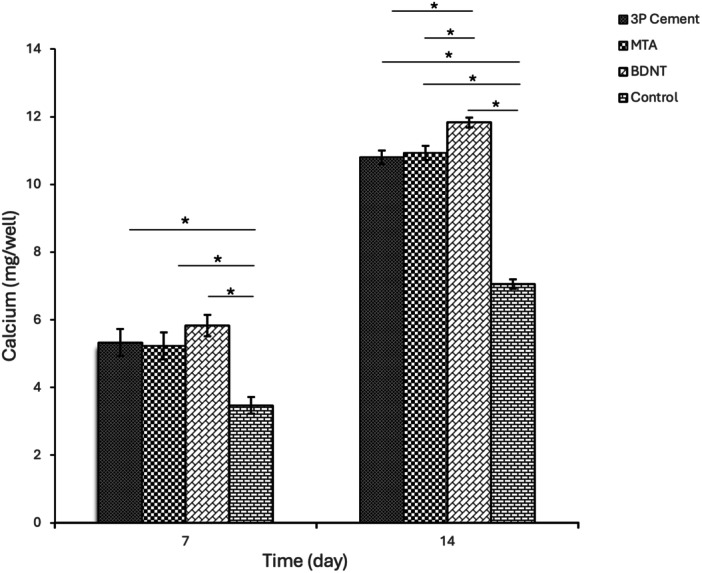
Mean calcium content with standard deviations and 95% confidence intervals for study groups on Days 7 and 14.

On Day 14, both ALP activity and calcium content remained significantly lower in the control group compared to the other groups (*p* < 0.001). ALP activity was highest in BDNT, significantly exceeding MTA (*p* < 0.05). There was no difference between MTA and 3P cement in ALP activity. BDNT demonstrated significantly greater calcium deposition than both MTA and 3P cement, which were comparable to each other (Figures 3 and 4 and Supplementary Tables S3 and S4).

#### 3.2.2 Alizarin Red Staining

Qualitative assessment using Alizarin Red staining revealed red‐stained mineralized nodules, indicative of osteogenic differentiation and calcium deposition by DPSCs.

**Figure 5:**
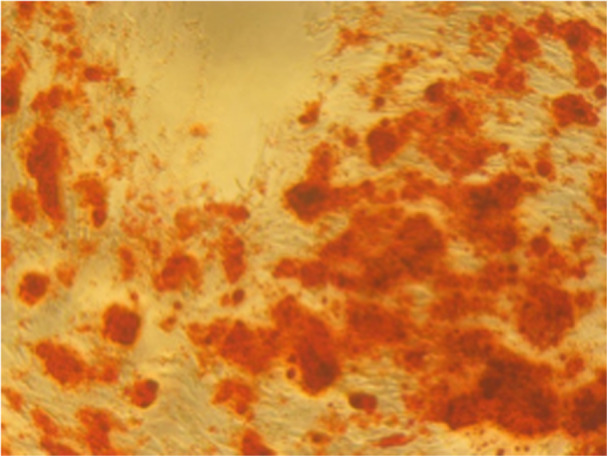
Alizarin Red staining of DPSCs cultured with 3P cement on Day 14.

**Figure 6:**
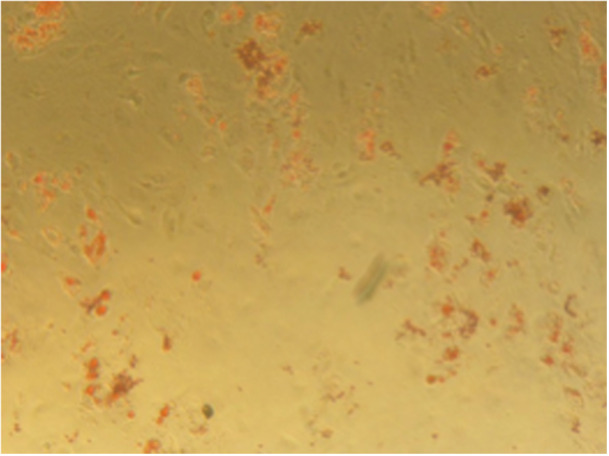
Alizarin Red staining of DPSCs cultured in the control group on Day 14.

On Day 14, dense and widespread mineralization was observed in the 3P‐cement group compared to sparse deposition in the control group (Figures 5 and 6).

## 4 Discussion

Bone defect reconstruction remains clinically challenging because the ideal graft substitute must combine bioactivity, predictable remodeling, and handling properties while minimizing morbidity and supply limitations. Given these practical constraints, synthetic ion‐releasing biomaterials have been increasingly explored as “instructive” matrices that modulate the local microenvironment to promote osteogenesis rather than merely acting as passive fillers (Al‐Rawee et al. 2023; Black et al. 2015; Oryan et al. 2014; Watering et al. 2012).

Accordingly, the present study focused on the in vitro biological performance of a previously characterized three‐phase cement (3P cement) composed of Sr‐HA, Si‐HA, and tricalcium silicate (C_3_S), using DPSCs as a clinically relevant progenitor cell source for craniofacial bone regeneration.

The physicochemical characteristics of the three‐phase cement have been previously reported by Parisay et al. (2022). In that study, SEM demonstrated distinct morphological features for each component, with calcium silicate exhibiting dense hexagonal crystals of submicron dimensions, Si‐HA showing elongated hexagonal crystals, and Sr‐HA presenting larger plate‐like structures. XRD analysis confirmed the formation of an apatite structure in the presence of silicon and strontium ions, while the silicate phase consisted of a mixture of dicalcium silicate and tricalcium silicate. These reported phase and morphology features provide a reasonable physicochemical context for the biological outcomes observed here, although they were not re‐measured in the current experimental setting.

Cytocompatibility outcomes suggested that extract concentration is a key determinant of DPSC response. The MTT assay and apoptosis analysis demonstrated that 3P cement was biocompatible and nontoxic, supporting the viability and proliferation of DPSCs over a 7‐day period. Notably, the most favorable profile was observed at the 1/5 dilution, where viability was highest, and apoptosis remained low, while the undiluted extract (Test 1) showed transiently higher apoptosis at early time points. This pattern is consistent with a concentration‐dependent “therapeutic window” often observed for ion‐releasing materials, where moderate ionic exposure supports cell function but higher exposure can induce cellular stress responses (Hoppe et al. 2011). In agreement with this observation, Siew Ching Hii et al. reported excellent DPSC attachment and biocompatibility when cultured with nano‐HA–silica glass ionomer cement, suggesting that silicon‐containing matrices can support early cellular adaptation when ionic exposure remains within a biologically tolerable range (Hii et al. 2019).

Such concentration‐dependent effects are well recognized for strontium in bone biology, including CaSR‐linked signaling effects that can promote osteoblast function while influencing osteoclast activity, with outcomes dependent on dose and context (Kołodziejska et al. 2021; Marx et al. 2020). Similar findings have been reported by Ehret et al. who observed that Sr‐containing matrices supported osteoblastic differentiation without inducing cytotoxicity across specific concentration ranges, further emphasizing the importance of dose in modulating cellular response (Ehret et al. 2017).

Beyond cytocompatibility, the ability of the material to promote osteogenic differentiation represents a more critical determinant of its regenerative potential. ALP is an early and critical marker of osteoblast differentiation, playing a pivotal role in initiating mineralization by hydrolyzing phosphate esters and increasing local phosphate concentrations (Fortuna et al. 1979; Tenenbaum 1987). In our study, DPSCs cultured on 3P cement exhibited a significant increase in ALP activity, which was accompanied by elevated calcium deposition, as confirmed by quantitative assays and qualitative Alizarin Red staining. Taken together, these endpoints indicate increased osteogenic‐associated activity and mineral deposition under the tested conditions. Abdel‐Rahman Youssef et al. similarly demonstrated increased ALP activity in DPSCs cultured with MTA and BDNT, both of which contain tricalcium silicate as a primary component (Youssef et al. 2019). This aligns with the broader observation that calcium silicate–based materials can support osteogenic responses, although their effects may vary with formulation and extract concentration. Consistent with this, Deog‐Gyo Seo et al. demonstrated favorable biological properties and enhanced mineralization activity of calcium silicate–based root canal sealers on DPSCs, reinforcing the relevance of CS‐containing systems in dental regenerative contexts (Seo et al. 2019).

Calcium silicate systems are frequently discussed as bioactive materials capable of releasing silicate‐related ionic cues that can influence osteogenic and angiogenic signaling; for example, silicate ions have been linked to pro‐angiogenic responses (e.g., VEGF‐related pathways) in relevant cell types (Dashnyam et al. 2017; Deng et al. 2017).

While the present findings suggest enhanced osteogenic‐associated activity in the 3P‐cement groups, the individual contribution of each phase was not isolated in this experimental design. Therefore, it is not possible to determine whether the observed biological response reflects additive effects or true synergistic interactions among Sr‐HA, Si‐HA, and C_3_S. Nevertheless, previous studies provide a mechanistic basis supporting the biological relevance of each component. Si‐doped HA has been reported to enhance osteoblast differentiation and ALP expression through the activation of Wnt/β‐catenin and MAPK signaling pathways (Sun et al. 2018). Sr‐doped HA similarly promotes osteogenic activity; for example, Guo‐Xin Ni et al. demonstrated that Sr incorporation into HA ceramic scaffolds significantly improved ALP activity and mineralized nodule formation (Ni et al. 2011). Moreover, Lixia Mao et al. reported that combined Sr and Si substitution in osteoporotic bone models improved regeneration outcomes, with Sr contributing to angiogenic and anti‐resorptive effects, while Si primarily supported osteoblast differentiation and matrix formation (Mao et al. 2017).

Building on these observations, the reported biological activity of strontium and silicon provides a plausible explanation for the trends observed in the present study. Strontium has been widely described as interacting with calcium‐sensing receptor (CaSR)‐associated signaling pathways in bone cells, with downstream effects that favor osteoblast differentiation while modulating osteoclastogenesis (Kołodziejska et al. 2021; Marx et al. 2020). Silicon substitution in apatite has likewise been associated with enhanced early osteogenic responses in vitro, including increased ALP expression, collagen synthesis, and matrix mineralization in several cell models. In addition, the calcium silicate phase (C_3_S) may further influence the local ionic environment through the release of calcium and silicate‐related species, which have been linked to bioactivity in similar cement systems.

Taken together, these mechanisms support the hypothesis that the composite formulation creates a concentration‐dependent ionic microenvironment capable of modulating DPSC proliferation and osteogenic activity. However, as noted above, the present experimental design does not allow the dissection of the individual or interactive contribution of each phase. Determining whether the formulation exerts additive or true synergistic effects will require future studies incorporating single‐component controls and defined combinations under identical conditions.

Interestingly, some studies have reported conflicting findings regarding the effects of Sr on bone metabolism. For instance, Wornham et al. found that high concentrations of Sr2+ salts acted as a general inhibitor of bone cell function, particularly mineralization (Wornham et al. 2014). These discrepancies further underscore the narrow therapeutic window associated with strontium exposure in bone‐related systems, as several studies have reported that low concentrations promote mineralization, whereas higher concentrations can inhibit this process (Guo et al. 2016; Li et al. 2019; Pasqualetti et al. 2013). Our concentration‐dependent pattern (better outcomes at higher dilution) is consistent with this broader dose‐sensitivity concept.

Overall, the present in vitro findings indicate that 3P‐cement extracts, particularly at the 1/5 dilution, support DPSC viability and are associated with enhanced ALP activity and mineral deposition compared with controls and reference materials.

However, this study has certain limitations. The present investigation employed a material extract model to evaluate cytocompatibility and osteogenic differentiation. While this approach allows the standardized assessment of soluble components released from the materials and is widely used for preliminary biological screening, it does not replicate direct cell–material contact, surface topography influences, or dynamic interfacial reactions occurring during the setting process. Therefore, the observed cellular responses primarily reflect the biological effects of material‐derived ions and leachable components rather than physical interactions at the cell–material interface.

Although the enhanced biological responses observed in the 3P‐cement groups may be associated with the presence of Sr and Si ions, the present study did not quantify ion release, pH variation, or osmolarity of the extracts. Therefore, a direct correlation between specific ion concentrations and cellular behavior cannot be definitively established. Future investigations incorporating inductively coupled plasma (ICP) analysis and physicochemical characterization of material extracts are necessary to elucidate the mechanistic relationship between ion release profiles and osteogenic stimulation.

While ALP activity and mineralization assays are widely accepted indicators of osteogenic differentiation, the present study did not include molecular evaluation of osteogenic gene or protein expression. Therefore, the observed enhancement in mineral deposition cannot be definitively attributed to specific osteogenic signaling pathways. Future investigations incorporating gene expression and protein analyses will be necessary to confirm activation of canonical osteogenic mechanisms and to further exclude nonspecific mineral precipitation.

In addition to biological evaluation, comprehensive physicomechanical characterization is essential for clinical translation of calcium silicate‐based scaffolds. Parameters such as setting time, compressive strength, washout resistance, handling properties, degradation kinetics, and apatite‐forming ability in simulated body fluid were not assessed in the present study. This is particularly relevant given the incorporation of CaSO_4_ as a porogenic component, which may influence early dissolution behavior and mechanical stability. Therefore, further studies integrating both biological and material performance analyses are required to fully determine the translational potential of the 3P cement.

Mineralization assays, such as ALP activity, calcium quantification, and Alizarin Red staining, are widely used indicators of osteogenic differentiation; however, these methods may be influenced by differences in cell number or nonspecific ionic precipitation. In the present study, normalization to DNA or total protein content and complementary mineral characterization techniques were not performed. Therefore, while the results indicate enhanced mineral‐associated activity, a definitive confirmation of structured extracellular matrix mineralization requires further investigation.

In addition, Alizarin Red S staining was primarily used as a qualitative indicator of mineral deposition through visual comparison between groups. Although clear differences in staining intensity were observed, quantitative extraction or spectrophotometric measurement of ARS was not performed. Therefore, while the findings suggest enhanced mineral‐associated activity in the 3P‐cement groups, the magnitude of mineralization cannot be precisely quantified. Future studies incorporating dye extraction assays and normalization to DNA or total protein content would provide a more robust confirmation of matrix mineralization.

As an in vitro experiment, it cannot fully replicate the complex biological environment of living bone tissue. Factors such as vascularization, mechanical loading, and immune response play critical roles in graft integration and long‐term stability, which cannot be adequately assessed in vitro (Salthouse et al. 2023). Future research should include in vivo animal studies to evaluate the material's biodegradation rate, angiogenic potential, and mechanical properties under physiological conditions. Additionally, further studies are required to characterize Sr and Si release profiles and determine concentration ranges that promote regenerative outcomes while minimizing potential adverse effects (Lu et al. 2024). Following confirmation of safety and efficacy in preclinical models, clinical trials will be needed to establish the material's performance in human patients and its suitability for routine clinical use (Jagadale et al. 2025).

## 5 Conclusion

In conclusion, incorporation of Sr and Si ions into a tricalcium silicate–based scaffold resulted in a cytocompatible material that supported DPSC viability and was associated with increased ALP activity and mineral deposition under in vitro extract conditions. These findings suggest that 3P cement possesses biologically relevant properties and warrants further investigation as a potential regenerative biomaterial. However, because the present results are derived from an extract‐mediated in vitro model, additional studies incorporating direct cell–material contact systems, quantitative ion‐release profiling, and in vivo evaluation are necessary to validate its biological performance and translational potential.

## Author Contributions

Nahid Nasrabadi conceived and designed the study. Ehsan Saburi, Atekeh Hosseinpour, and Hossein Bagheri performed the experiments and collected the data. Ehsan Saburi and Hossein Bagheri contributed to materials preparation and technical support. Pooya Saeedi and Mahsa Ghorbani analyzed the data and drafted the manuscript. Pooya Saeedi supervised the study and critically revised the manuscript. All authors reviewed and approved the final version.

## Ethics Statement

The ethics committee of the Mashhad University of Medical Sciences granted approval (IR.MUMS.DENTISTRY.REC.1398.114).

## Consent

The authors have nothing to report.

## Conflicts of Interest

The authors declare no conflicts of interest.

## Consent for Publication

The authors have nothing to report.

## Supporting information

Supporting File 1

Supporting File 2

Supporting File 3

Supporting File 4

## Data Availability

Data supporting the findings of this research are available upon reasonable request from the corresponding author.
