# Supplementary material for: Osteogenic Potential of a Three‐Phase Strontium‐ and Silicon‐Doped Tricalcium Silicate Cement on Dental Pulp Stem Cells: An In Vitro Study
Source: Clin Exp Dent Res. 2026 May 3;12(3):e70362. doi: 10.1002/cre2.70362 (PMC13135791; doi:10.1002/cre2.70362)
Supplement: Supplementary file 1 — Supporting File 1 [file CRE2-12-e70362-s001.docx]

Supplementary Table S1. MTT assay results showing cell viability of DPSCs cultured with different material extracts at days 1, 3, and 7. Data are presented as mean [standard deviation] from three independent experiments.

| **Group** | **Day 1** | **Day 3** | **Day 7** |
| --- | --- | --- | --- |
| Test 1 | 99.840 [2.668] | 101.622 [1.833] | 109.831 [5.338] |
| Test 1/2 | 104.807 [3.802] | 107.906 [3.082] | 117.099 [4.591] |
| Test 1/5 | 108.574 [1.303] | 116.031 [7.555] | 127.347 [8.137] |
| MTA | 83.276 [2.075] | 92.890 [3.955] | 99.935 [7.666] |
| Positive Control | 0.562 [0.073] | 0.760 [0.179] | 0.625 [0.036] |
| Negative Control | 100.000 [0.000] | 119.667 [2.517] | 126.667 [3.786] |

**Note:** Cell viability values are expressed as percentages relative to the negative control group at Day 1 (set as 100%).
