# Supplementary material for: Osteogenic Potential of a Three‐Phase Strontium‐ and Silicon‐Doped Tricalcium Silicate Cement on Dental Pulp Stem Cells: An In Vitro Study
Source: Clin Exp Dent Res. 2026 May 3;12(3):e70362. doi: 10.1002/cre2.70362 (PMC13135791; doi:10.1002/cre2.70362)
Supplement: Supplementary file 2 — Supporting File 2 [file CRE2-12-e70362-s004.docx]

Supplementary Table S2. Percentage of total apoptotic cells determined by Annexin V-FITC/PI flow cytometry at days 1, 3, and 7. Data are presented as mean [standard deviation] from three independent experiments.

| **Group** | **Day 1** | **Day 3** | **Day 7** |
| --- | --- | --- | --- |
| Test 1 | 9.450 [1.061] | 8.450 [0.919] | 4.175 [0.884] |
| Test 1/2 | 5.165 [0.502] | 4.700 [0.566] | 3.700 [0.566] |
| Test 1/5 | 3.055 [0.092] | 4.040 [0.198] | 3.950 [0.212] |
| MTA | 4.845 [0.191] | 5.600 [0.075] | 6.050 [0.636] |
| Negative Control | 1.350 [0.495] | 1.710 [0.834] | 1.450 [0.495] |
