# Supplementary material for: Osteogenic Potential of a Three‐Phase Strontium‐ and Silicon‐Doped Tricalcium Silicate Cement on Dental Pulp Stem Cells: An In Vitro Study
Source: Clin Exp Dent Res. 2026 May 3;12(3):e70362. doi: 10.1002/cre2.70362 (PMC13135791; doi:10.1002/cre2.70362)
Supplement: Supplementary file 3 — Supporting File 3 [file CRE2-12-e70362-s003.docx]

Supplementary Table S3. Alkaline phosphatase (ALP) activity (IU/mg total protein) of DPSCs cultured with different material extracts at days 7 and 14. Data are presented as mean [standard deviation] from three independent experiments.

| **Group** | **Day 7** | **Day 14** |
| --- | --- | --- |
| 3P Cement | 0.90 [0.02] | 1.92 [0.05] |
| MTA | 0.91 [0.03] | 1.80 [0.02] |
| BDNT | 1.06 [0.06] | 2.02 [0.07] |
| Control | 0.63 [0.05] | 0.87 [0.02] |
