# Supplementary material for: Osteogenic Potential of a Three‐Phase Strontium‐ and Silicon‐Doped Tricalcium Silicate Cement on Dental Pulp Stem Cells: An In Vitro Study
Source: Clin Exp Dent Res. 2026 May 3;12(3):e70362. doi: 10.1002/cre2.70362 (PMC13135791; doi:10.1002/cre2.70362)
Supplement: Supplementary file 4 — Supporting File 4 [file CRE2-12-e70362-s002.docx]

Supplementary Table S4. Calcium deposition (mg/well) of DPSCs cultured with different material extracts at days 7 and 14. Data are presented as mean [standard deviation] from three independent experiments.

| **Group** | **Day 7** | **Day 14** |
| --- | --- | --- |
| 3P Cement | 5.33 [0.40] | 10.80 [0.20] |
| MTA | 5.23 [0.40] | 10.93 [0.21] |
| BDNT | 5.83 [0.31] | 11.83 [0.15] |
| Control | 3.47 [0.25] | 7.06 [0.14] |
